# Origins, production, and utilization of cassava in Burkina Faso, a contribution of a neglected crop to household food security

**DOI:** 10.1002/fsn3.408

**Published:** 2016-07-20

**Authors:** Flibert Guira, Koussao Some, Donatien Kabore, Hagrétou Sawadogo‐Lingani, Yves Traore, Aly Savadogo

**Affiliations:** ^1^ Laboratory of Applied Biochemistry and Immunology (LabIA); Department of Biochemistry‐MicrobiologyUniversity Ouaga I Professor Joseph KI‐ZERBO; ^2^ National Institute of Environment and Agriculture Research (INERA/CNRST)OuagadougouBurkina Faso; ^3^ Laboratory of MicrobiologyFood Technology Department (DTA/IRSAT/CNRST)OuagadougouBurkina Faso

**Keywords:** cassava origin, household food security, processing, production, varieties

## Abstract

Cassava (*Manihot esculenta* Crantz) is a food plant introduced in Africa from America by the Portuguese in 1558. The objective of this study is to establish cassava origins, production, and utilization in Burkina Faso. The investigation was carried out in the regions of Center West, Cascades, Boucle du Mouhoun, Hauts Bassins, South West, and Center East of Burkina Faso. Eighteen cassava processing units and 226 farmers in 57 communities from the selected regions have been involved in the survey. The investigation showed that cassava was introduced to Burkina Faso, former Upper Volta from the costal countries, Gold Coast (now Ghana), by both local traders and the Roman Catholic White missionaries. This happened between the second half of the 19th century and the beginning of the 20th century. The main variety introduced was Banfti. Some improved varieties like V5 (94/0270), Banké (V2), 68.61, 30572, KTMA developed by research are now available and used by farmers along with the traditional varieties like manchien, santidougou, tchinda yaar, léo. The cases of intoxication evoked by some farmers are evidence that some of those varieties may have a high level of cyanohydric acid content. Cassava is available all the year throughout the country. But the top of cassava production is reached in July. Most of the small‐scale farmers (98%) grow cassava both for household use and as income generator. About 83.92% of cassava farmers have less than 10 tons as annual production and only 1.72% of them can produce more than 100 tons. The main food products based on cassava found in communities are raw roots, boiled roots, roasted roots, tô, attiéké, tapioca, ragout, beignets, boiled leaves, soup (with leaves), cassava juice, etc. And the main cassava‐processed products in the processing units are attiéké, gari, tapioca, and flour. Cassava contributes greatly to household food security during food shortage period. It sustains families for weeks as food and is also exchanged with other foods or sold to buy food or meet household needs.

## 1 Introduction

Cassava (*Manihot esculenta* Crantz) is a food plant brought from the New World to the Tropical Africa where it does now establish (Roger, 2014). The utilization of cassava as food for America's societies began around the 18th century before Christ (Charrier & Lefrèvre, 1994). It is a woody plant with about 1–3 m height belonging to Manihot gender and Euphorbiaceous family (Bombily, 1995). There are hundreds of cassava varieties spread around the world (Favier, 1977; Laure, Pinton, & Sécond, 1998). Approximatively the half of the world's cassava current production comes from Africa where it is cultivated in around 40 countries.

Cassava is the third most important source of calories in the tropics after rice and maize (Food Safety Network, 2014). Its processed products contain an important proportion of carbohydrates (mainly starch) and minerals (Guira, 2013). Cassava leaves contain protein, vitamins (A and C), and a lot of mineral salts (Ravindran & Ravindran, 1988). Cassava is cultivated both as food (for human and animals) and as industrial raw material (FAO, 2012). The most important industrial utilizations of cassava are ethanol, starch, biofuel, flour, biscuits, bread, jelly, thickening agents, gravies, custard powders, babies’ food, glucose, and confectioneries (Echebiri & Edaba, 2008). The main cassava food found in Africa are attiéké, tapioca, gari, flour, starch, futu, fermented flours, akpissi, alebo, eberebe, ragout, kwadu, ground fresh tuber, kenkey, fede, agbelilakia, placali, yakayake, cossette, lafun, chikwangue, etc. (Andrew, 2002; Echebiri & Edaba, 2008; Younoussa, Momar, Mama, & Praxéde, 2013). Cassava generates billions of income both for families and government and then contributes a lot to food security at several levels (FAO, 2012).

In Burkina Faso, cassava has been introduced some decades ago. It used to be an adequate solution to household food security during food shortage period in some regions. The fermentation technology incorporated in the population's food habits is increasing cassava production and its utilization as food. This contributes to the development of cassava processing units. Cassava is now cultivated both during rainy season and dry season under irrigation. Several improved varieties are available and adapted to the national agroecological conditions and the processing technology. Attiéké is the most widely consumed cassava‐based product in Burkina Faso. Its production contributes in generating incomes especially for women (Diacoumba, 2008). The aim of this study is to establish the origins of cassava varieties grown in Burkina Faso, to evaluate cassava variety knowledge and use, the state of its processing, and its contribution to household food security.

## 2 Materials and Methods

An investigation was carried out in the main cassava production areas of Burkina Faso. The targeted regions were Center West, Cascades, Boucle du Mouhoun, Hauts Bassins, South West, and Center East. Cassava processing units and cassava producers were both the target of the investigation. About 226 farmers from 57 communities in the selected regions were involved in this investigation for cassava production and use of cassava by the households. The data were collected using a questionnaire by SphinxV5demo software. The questions included knowledge of cassava origin in the locality, cassava varieties used or known, the production level, cassava production challenges and opportunities, it family use, and the knowledge about cassava toxicity. For the industrial uses and cassava processing, another questionnaire was used. Eighteen processing units using cassava roots as raw material and located in Léo, Dédougou, Gaoua, Kampti, Orodara, and Bobo Dioulasso were surveyed. The required questions focused on the processing level and cassava‐based products. The collected data were then analyzed with R statistical software for average determination variance and linear regression. The graphics are made with Excel (2010).

Food security definition here used is base on the FAO (2012) recommendation of 2300 kcal per adult per day to evaluate the contribution of cassava to household food security. Food security status of households can then be expressed nationally as follows:

(1) $$F_{\mathrm{secn}}=0\;\;\text{for}\;X_{n}<Z$$

(2) $$F_{\mathrm{secn}}=1\;\;\text{for}\;X_{n}>Z$$

where *F*_secn_ = food security status of household n; *X*_n_ = per capita calorie consumption of household n; *Z*_n_ = recommended daily minimum energy requirement of 2,300 kcal.

The contribution of cassava to family need is evaluated based on equation (3) of household needs:

(3) $$\begin{array}{cc} N(\mathrm{Needs})\;=\; & \text{HP(Household Production)}-\text{L(Losses)}\\ & \pm \text{ST(stocks)}-\text{S (Sale)}+\text{P(Purchase)} \end{array}$$

## 3 Results

### 3.1 Origin and spread of cassava in Burkina Faso

The investigation shows different routes of cassava introduction in Burkina Faso. It varies according to region. In Center West region, all interviewed person recognize two ways of cassava introduction. The Roman Catholic White missionaries introduce cassava for the first time in Sanguie province three or four generations ago. They do not know the exact year. The local traders also introduce it in Sissili areas from Gold Coast, now Ghana. But in the early end of the 20th century, some other varieties from Côte d'Ivoire were introduced and cultivated first in the western part of the country along with the older variety. In Cascades, Boucle du Mouhoun, and Hauts Bassins regions, cassava was introduced by both government and migration movement especially from Côte d'Ivoire. The government introduced it from zones where the crop was grown and from demonstration sites. And the favorite sites of its cultivation were irrigated places. The irrigation project of Samadeni dam allowed experimentation of cassava growth around year 1987 in Hauts Bassins region. For many interviewed persons, the Ivorian political crisis in 1998 during which many Burkinabe have been pushed back to their homeland stimulated cassava production and processing in the western part of the country. In the Center East region, cassava was introduced by local traders (from Ghana) and also by farmers from other localities. In South West region, cassava was introduced from Côte d'Ivoire. The main factors that increased cassava production in a larger scale in this region were due to a German‐based Agriculture Development Program (PDA) and the government actions at the early beginning of the 21th century.

### 3.2 Cassava production and available varieties in Burkina Faso

**Figure 1:**
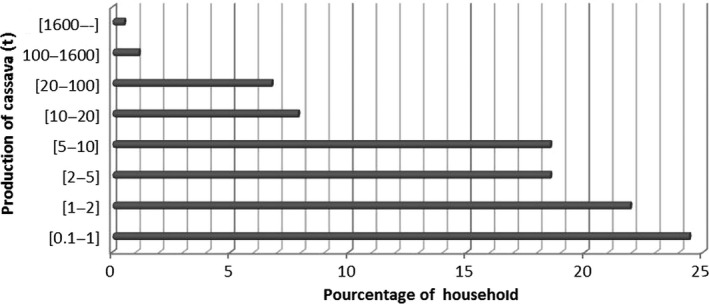
Estimation of cassava production level in relation to the proportion of households involved

Till date, cassava in not registered as national food plant by agricultural statistic division of the government. The evolution of its annual production is still unknown. The production of cassava throughout the country was 60,000 tons in 2013 as estimated by the PDA technicians. In general, cassava is cultivated in a small scale for family consumption and sold or exchanged with other items during food shortage period. The proportion of cassava producers according to the level of their production is as shown in Figure 1. Importantly, it appears that cassava farmers have less than 10 tons as annual production. Only 1.72% of them have more than 100 tons as annual production. Cassava is then produced in small scale essentially for family use.

**Figure 2:**
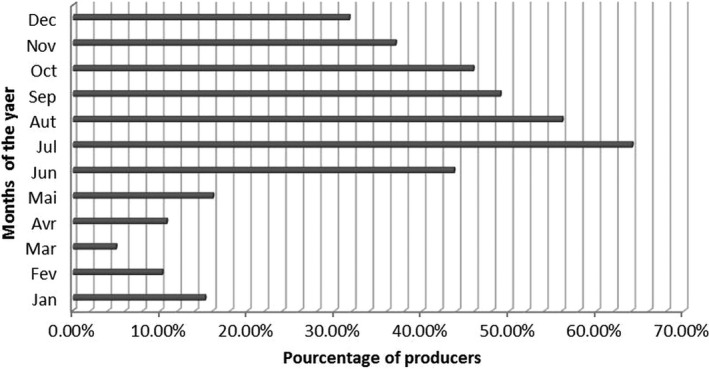
Availability of cassava during a year in Burkina Faso

**Figure 3:**
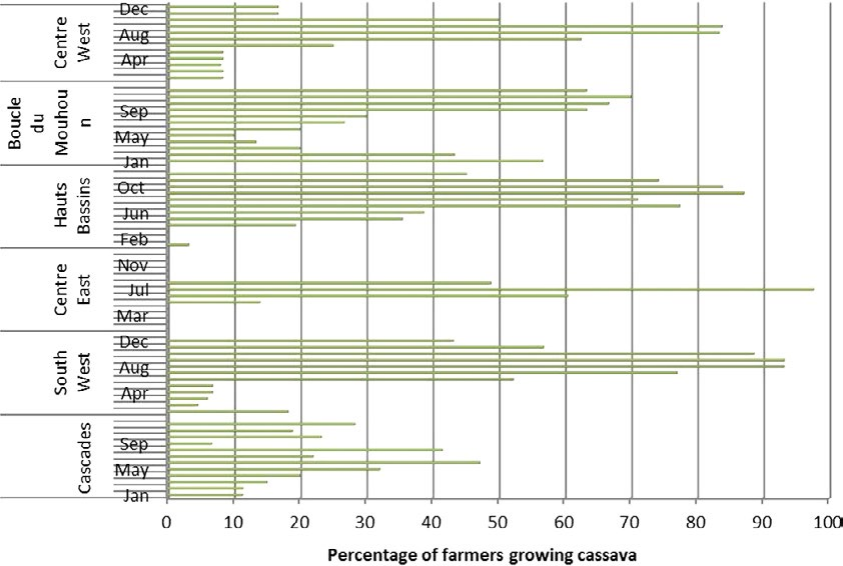
Availability of cassava during a year according to the regions in Burkina Faso

The availability of cassava in Burkina Faso varies from one region to another according to the variety used and the possibility to produce it during the dry season (Fig. 2 and 3). Improved cassava varieties are in full diffusion from South West region to Center East region where it has been tested first. But farmers are still attached to their traditional varieties for family use. Improved cassava varieties are better for processing, but not ready to be eaten. The availability of cassava in Burkina Faso is as shown in Figure 2. The cassava production is high in July and more than 40% of its farmers have the availability from June to October.

Figure 3 highlights the fact that the availability of cassava in Burkina Faso depends on the period of the year, the main period of production is ranged between June and October. The dry season is characterized by a small quantity of cassava root available. According to the region, there is a variation in cassava availability. In Center East region, cassava is available only during the rainy season. In the Hauts Bassins, Cascades, and Boucle du Mouhoun regions, cassava is available all the year (Fig. 3).

The characteristics of cassava varieties found during the survey are as shown in Table 1. Many cassava varieties are cultivated in Burkina Faso. Some of these varieties are from Côte d'Ivoire (Santidougou local variety, Bounoua, Bounoua griguiti, Manioc ivoirien, KTMA), some from Ghana (Banchii/Manchien, Sauvage cassava, Léo local variety, Tchinda Yaar), and other are national improved varieties from research institute mainly INERA (94/0270, Banke, 30572, 68.61). Cassava varieties are chosen according to the duration of their production cycle, their sweetness, and the mineral composition of the soil where it is cultivated. The toxicity of cassava varieties is subjective; some farmers considered some varieties as toxic, while some considered it as nontoxic. But 59.46% of household had a case of cassava intoxication. Households use drying and soaking as processes to eliminate cassava toxicity. The cassava varieties identified in this study are shown in Table 1.

**Table 1:** Identified cassava varieties in Burkina Faso

Appellations	Variety names	Locality	Cycle(Months)	Average of stalkheight (m)	Leaves morphology	Root and others characteristics	Toxicity(according to farmers)
Banchii, Manchien	Banfti	Centre West (Sanguié)	12–18	0.5–02	Red leafstalk	Big tubers, threadlike, white, explode to cooking	No
Santidougou local variety	NIV^a^	Hauts Bassins (Santidougou, Desso)	09–12	2–3	Red leafstalk	Big and long	No
V5 variety	(94/0270)	Boucle du Mouhoun, Hauts Bassins	05–10	2–3	Leaves brought closer, dark color, middle sizeCloser's leaves, dark green color, middle size	Dark color, cooking difficult, big tuber, good output, not ready to eat	No/Yes
KTMA variety	KTMA	Hauts Bassins	09–12	1.2–02	Fattening green color, more lobe’ leaves	No	No
Bounoua (White cassava)	Bounou	Centre West,Cascades, South West	05–06	1–2.5	Red leafstalk, large leaf	Big and long	No
Manioc ivoirien	NIV^a^	South West	06–10	1–3	Large leaf, green leafstalk	Soft, good cooking, few fibers	No
Bounoua griguiti	Bounoua	South West	08–10	0.5–2	Middle size of leaf	Short, big and slightly reddish	Yes/No
Tchinda Yaar (Zabré local variety)	Banfti	Centre East	1.2–5		Big, white phéloderme		No
Variety 68.61	68.61	South West	08–09	0.5–2	Little size of leaf	Sandy soil, not too humid	No
Banké (V2)	V2	Boucle du mouhoun, South West	10–12	1–2.5	large leaf	Threadlike, whitish	No
Sauvage cassava	NIV^a^	South West, Centre West	12–18	1–3	Large leaf, green leafstalk	Big and threadlike to hard and thick skin	No
Variety 30572	30572	South West	06–08	0.5–2	Middle size of leaf	Big tubers	Yes/No
Léo local variety	NIV^a^		09–15	0.5–2	Large leaf, red leafstalk		Yes

a NIV: Note Identified Variety (the appellation and descriptions are not sufficient to specify the variety it belongs to).

### 3.3 Cassava utilizations at household level in Burkina Faso

**Figure 4:**
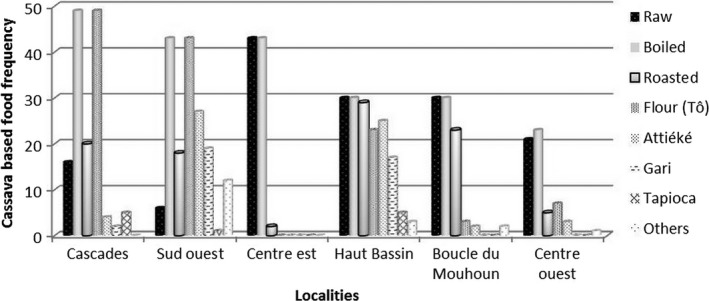
Diversity of cassava food products according to the locality

Cassava cultivated in Burkina is used essentially for human food. Animals are fed only with cassava leaves and cassava root peels and sometime mixed with salt. In the communities, cassava is mainly produced to reduce food shortage period in addition to nonwood forest products. The most consumed cassava‐based food in Burkina Faso is the boiled roots. The main cassava‐based foods identified in villages according to their frequencies in households are boiled roots (96.90%), raw roots (64.90%), cassava flour (55.60), roasted root (43.10%), attiéké (27.10%), gari (16.90%), and other cassava products (8%). These results highlight the fact that cassava‐manufactured food and cassava‐fermented food are not well established in household food habit. Cassava is cultivated not for processing, but mainly for daily use during food shortage period. The relationship between the diversification of cassava‐based food and the locality is shown in Figure 4. In the regions of Hauts Bassins, South West and Cascades, peoples have knowledge about cassava based food. Consequently, people diversified their cassava based food at home. Boiled root of cassava is the most consumed in Burkina Faso, particularly during the rainy season. During rainy season, farmers have a lot of work to perform. They use efficient methods to make their food ready.

### 3.4 Utilization of cassava in processing units in Burkina Faso

**Figure 5:**
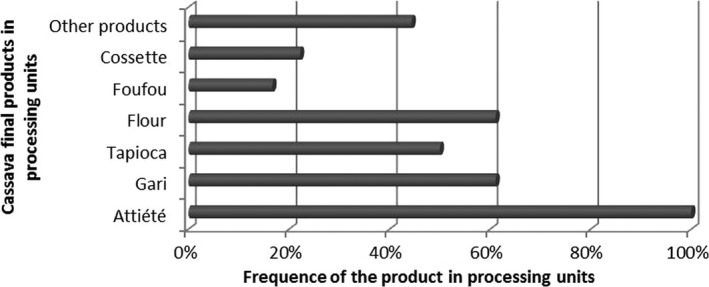
Cassava processed products in processing units in Burkina Faso

Figure 5 shows the cassava utilization in processing units. The number of cassava processing units is estimated to be 437 in Burkina Faso, but about 20 of them are semimechanized. Cassava processing in Burkina Faso is very recent. About 83.33% of processing units are less than 16 years old. Cassava processing started just after the political crisis occurred in Côte d'Ivoire in 1998. Families back from Côte d'Ivoire were supported to facilitate their economic integration. The women were particularly trained in cassava processing and they are now leaders in cassava processing. The producers are organized in association (44.4% of units) or private group (55.6% of units). More than 50% of the processing units employ more than 12 workers and the greatest unit has 60 workers. About 73.33% of units have less than 200 tons of cassava as raw material for their annual production, and 20% of them have 300–400 tons of cassava as annual raw material. Only 6.67% of the units have more than 600 tons of cassava as annual raw material. The most processed products of cassava in the units are attiéké, gari, tapioca, flour, foufou, cossette, and other products such as starch, fermented flour, curds, etc. Attiéké is the most processed product, found in all the units. The regions of Hauts Bassins, Cascades, and South West in Burkina Faso are those where cassava is processed mostly. The final products of cassava are sold in local markets and also exported to Mali republic and even to Senegal.

**Figure 6:**
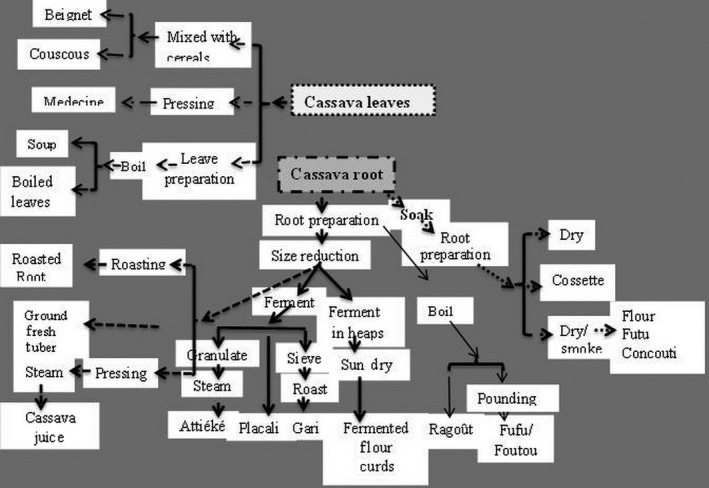
Interrelationship of cassava products from 57 villages in Burkina adapted from Henry et al. (1998)

Cassava leaves are sometime used as medicine, mixed in cereals to produce various meals. Many processes are used to prepare cassava‐based food. The interrelationship of cassava‐based products found in all the investigated communities is shown in Figure 6.

## 4 Discussion

### 4.1 Cassava origins and spread in Burkina

The investigation shows that cassava was introduced in Burkina Faso (Upper Volta) for the first time from Gold Coast (Ghana). Cassava introduction in Burkina Faso is very late compared to the coastal countries (Hillocks, 2002; Eke‐Okoro & Njoku, 2012) and occurred during the beginning of the 20th century. This statement is in accordance with the cassava varietal spread study in Africa (Adams, 1957). The two principal paths through which cassava was first imported to Burkina are the local traders and the Roman Catholic White missionaries. Other movements like migration (from Côte d'Ivoire) and internal distribution movement spread cassava throughout the country.

The local traders are the first to introduce cassava in the country. Some tribes in Burkina (mainly Mossi and Bissa) were involved in many trades in Gold Coast (Ghana) during the 17th and 18th centuries (Rouch, 1956). They used to sell cotton strips, livestock, and slaves in Gold Coast (Ghana). And they brought their salt and cola to be sold in their homeland. During the beginning of the 20th century, the European's occupation secured the roads and then increased this trade to a great extent (Rouch, 1956). At the same time, cassava was an appreciated crop widely spread (since the second half of the 18th century) throughout Ghana (Adams, 1957). The traders who found this new crop brought it back to Upper Volta (Burkina Faso) for experimentation. This was the first introduction of cassava in the country. It happened between the second half of the 19th century and the beginning of the 20th century. The exact date (year) is still unknown. The targeted localities were only the southern, the eastern, and the Center East part of the country.

Roman Catholic White missionaries also introduced cassava in Réo township (Sanguié province in the Center West part) around 1912 during the foundation of their mission agency there (Hououdié, 2008; Appolinaire, 2012). The general district of this mission agency was established in Tamale in Ghana where cassava was already promoted (Tiendrebeogo, 2008). It is from Tamale that the missionaries bring cassava to Burkina Faso. Réo and the surrounding villages began to experiment this new crop which was very profitable. In the last half of the 20th century, Sanguié was the most producing area of cassava in the country.

From these initial areas, cassava widely spread throughout the country. Some other cassava varieties like bounoua, banket, grigriti, etc., were imported from Côte d'Ivoire during the early end of the 20th century to the southern and western part of the country. The development of some dam between 1984 and 1987 contribute to spread and increase cassava and other market crops production in many areas. The national irrigation project developed between 2002 and 2007 during dry season to overcome food insecurity also associated cassava as profitable food plant. And it contributes in cassava spreading. The improved cassava varieties distributed increased the production considerably (Sidwaya, 2014). But the most important movement of cassava varieties is between farmers of the same locality and across regions. Nowadays cassava is found in many localities in Burkina Faso, from the southern to the northern and from the western to the eastern part of Burkina Faso. Cassava is cultivated mostly by poor farmers (96%) in their gardens to be used during food shortage period and also to increase their income. The money they got is usually used for school fees.

As in some African tropical countries, cassava production and use are still increasing in Burkina Faso communities. But in other countries, cassava is cultivated some centuries ago and it is now highly industrialized. According to the standard study of cassava in Africa (Jones,^1959^; Lebot,^2003^), the first reference of cassava in African’ continent was in 1558. In West Africa (Ghana), the Portuguese grew the crop around their trading ports, forts, and castles and it was a principal food consumed by both Portuguese and their slaves. The Portuguese are responsible of the initial diffusion of cassava around the world (Jones, 1959; Nweke & Lynam, 1997; Hillocks, 2002; IFAD and FAO, 2005; Chavarriaga‐Aguire & Halsey, 2005; Eke‐Okoro & Njoku, 2012; Michael et al., 2013).

### 4.2 Cassava production and available varieties in Burkina Faso

Cassava name varies according to the language. It sometime takes a designation of it derivate food's name. Cassava alternative names are mandioka (Portuguese, from Tupian), rogo (Hausa), mbay (Fulfulde), ege (Yoruba area), and banakun in Mande and Dogon area (Roger, 2014). The first cassava variety introduced in Burkina is Bantfi (Roger, 2014). From this variety name (Bantfi) derived the local alternative names of cassava in the country: manchien (Gourounsi), bandakou (Mossi), mandakou (Bissa), etc. The criteria used in this identification of cassava varieties were based on farmers’ descriptions (IFAD and FAO 2005; Manusset, 2006). These descriptions did not take into account the agronomical characteristics of these varieties (ITRAD, 2009). Thirteen cassava varieties were identified in all the investigated communities. The improved cassava varieties are more and more cultivated. There is a need of chemical analysis to certify the toxicity of the identified varieties. Despite the fact that farmers are the most important in Burkina (80% of the active population) (OCHA, 2014), a few number of them are involved in cassava production. About 83.92% of cassava farmers have less than 10 tons as annual production and 24.41% of them have less than 1 ton. Only 1.72% of cassava farmers have more than 100 tons as annual production. About 1% of cassava farmers cultivate it only for sale. In Burkina Faso, cassava is generally available during the rainy season. The availability varies according to regions and depending on the availability of irrigation system.

Cassava is a neglected crop in Burkina Faso (Diacoumba & Gantoly, 2008). The implication of research in cassava improvement is recent comparatively to other countries. The sweeter varieties are the most spread and only 0.45% of cassava farmers use the bitter one. But the nature of cassava variety is not the only factor of its bitterness. The minerals soil composition and the practices during cassava cultivation can lead to a bitter taste. About 59.46% of household had a case of cassava intoxication. The identified symptoms of this intoxication are stomach aches, distending, insomnias, vomiting, dizziness, jolts, diarrhea, constipation, tiredness, headaches, and paralysis. Some of these symptoms are clinically proved as cassava intoxication (Délange & Ahluwalia, 1982; Hovette et al.,^1992^; Hugon, 1998; Bennito & Silvana, 2010). Drying and soaking are the main processes used by household to eliminate cassava toxicity. The criteria of adoption of cassava depend on its sweetness as food and its ability to be sold in the market. Cassava is a promoting crop, but the greatest challenges of its production are the domestic animal that devastate the plant (60.4%), the insufficiency of water (43.6%) during the dry season, and the lack of specific fertilizer for cassava. The main factors limiting its long‐time use is its perishability.

Burkina Faso is among the countries where cassava production is not important (Okibgo; 1980) cited by Felix (2004). In 1998, the production of cassava in the country was estimated to be 2,000 tons by FAO (Hillocks, 2002). The production increased during this decade to reach to 60,000 tons (in South West and Center West) in 2013 according to PDA technicians interviewed. Burkina Faso is not considered as cassava production country in West Africa. The annual production of cassava in all African countries is estimated to be 153 million tons in 2012 and the production in Nigeria, the leader in African cassava production, is estimated to be 57 million tons in the same year (FAO, 2012). The main crop produces in Burkina Faso is sorghum and the research on cassava for industrial use is still not significant comparatively to other countries (Johanna, 1998; Rusika, Mahun, Jumbo, Sandifolo, & Malindi, 2010; Ademola, Ogugua, & Parayil, 2012; Eke‐Okoro & Njoku, 2012; Michael et al., 2013). Research on cassava is still insufficient to provide significant number of improved cassava varieties for different uses (Oluwole, Onabolu, & Mtunda, 2007; Eke‐Okoro & Njoku, 2012). In coastal countries there are many recipes made from cassava than in Burkina Faso (Andrew, 2002; FAO and IFAD (2005); Tolly, Lolo, & Emmanuel, 2013; Younoussa et al., 2013). Cassava leaves are very appreciated in some areas. Cassava leaves have a nutritive value similar to other dark green leaves and are an extremely valuable source of vitamin A (carotene) and vitamin C, iron, calcium, and protein (Lathan, 1979) cited by (IFAD and FAO 2005). Cassava leaves consumption helps to compensate the lack of protein and vitamins in cassava roots. The industrial use of cassava for starch, ethanol, biofuel, bread, etc., is not started yet in Burkina Faso (Felix, 2004; IITA and FAO 2005).

### 4.3 Contribution of cassava to household security

Since 1970, food insecurity used to be recurrent in Burkina Faso every year. It occurred early in the rainy season and last until the next crop harvest period. It usually happened from March to October (Janin, 2003; Thombiano, Natacha, Lamien, & Didier, 2012). It is a critical time when farmers sale their properties to acquire food. Many strategies like nonwood forest products consumption, diversification of agricultural production, etc., were undertaken to overcome food insecurity in this period. Cassava production is one of these alternatives. But its production and consumption is lower (0.3%) compared to costal countries like Cote d'Ivoire, Togo, Ghana, and Nigeria. Cassava is mainly cultivated by households to reduce food shortage period. At this time, cassava contributes to food availability, its stability, and accessibility.

In equation (1), food security is zero because households in the category have per capita calorie consumption (Xn) less than the recommended minimum of 2,300 kcal per adult per day. Households represented in equation (2) are those that have per capita calorie intake of more than or equal to this recommended minimum requirement. During food shortage, many households are in the case of equation (1). They lack food stock. The average of cassava production in households is around 1 ton, 399 kg according to the dry matter. The energetic contribution of cassava to household food security is about 1,596,000 kcal. And according to Ouédraogo et al., (2007), the average of household member's numbers is about 11 in rural areas. The cassava production of about 1 ton can contribute to satisfy the energetic requirement of the household for 63 days if it rationally used.

According to equation (3), cassava contributes to household needs by increasing household production and stocks and by reducing food purchase level during shortage time. Cassava is also given to other families as local food aid. This contributes to secure some household food security for a short time. In addition to family use, cassava is also exchanged with other food supplies. The exchange increases the accessibility of households to other food for their daily food balance. Cassava processing and cassava sell generate an important income for households. But this income is not only used to buy food, but also help to cover the household other needs. More than 40% of cassava farmers use the money they got from cassava selling to pay school fees for their children.

In some regions, cassava processing creates jobs for hundreds of people which generate income for household. During food shortage period, the income is mainly used for food need. This indirect contribution of cassava to household food security is also significant for families.

## 5 Conclusion

This study establishes proofs of cassava introduction in Burkina Faso during the beginning of 19th century. It spread out throughout the county. Cassava is now one of the nutritious crops in some regions and gradually imposes itself as a cash crop nationwide. Many cassava varieties, including improved one, are now in diffusion in the country. Cassava improved variety are essentially located in areas where institutes are promoting it. Improved cassava varieties have short cycle of production. But, they are not really use in families. From the root mush consumption, the utilization of cassava as food also knew a technological and notably a nutritional progress by the process of fermentation. It is now a raw material for a full flight food industry. The most interesting role of cassava is its contribution to household food security especially during food shortage period. The survey highlight the out‐flow problem for cassava. A follow‐up of both farmers and producers and research support is needed to improve cassava production and utilization in Burkina.

## Funding Information

No funding information provided.

## Conflict of Interest

None declared.
